# Impact of preoperative femoral vein ultrasound localization on puncture success and procedural complications during radiofrequency catheter ablation for atrial fibrillation: A multicenter study

**DOI:** 10.1097/MD.0000000000048511

**Published:** 2026-05-01

**Authors:** Xiaobo Liao, Qiao Xiao, Xueyuan Guo, Yunong Li, Xin Chen, Longjiang Hu, Xin Su, Lan Ren, Fangbing Wang, Peng Xiao

**Affiliations:** a Department of Cardiovascular Medicine, FuLing Hospital Affiliated to Chongqing University, Chongqing City, China; b Department of Cardiovascular Medicine, The Central Hospital of Yueyang, Yueyang City, Hunan Province, China; c Department of Arrhythmia Center, Beijing Anzhen Hospital, Capital Medical University, Beijing City, China; d Department of Cardiology, Zhangjiakou First Hospital, Zhangjiakou City, Hebei Province, China.

**Keywords:** arteriovenous fistula, atrial fibrillation, complications, femoral venous puncture, pseudoaneurysm, radiofrequency catheter ablation, ultrasound localization, uninterrupted anticoagulation

## Abstract

Safe and accurate femoral venous access is fundamental to the success of radiofrequency catheter ablation (RFCA) for atrial fibrillation (AF), particularly under uninterrupted anticoagulation, where vascular injury may lead to amplified bleeding and thrombotic risk. Conventional landmark-based puncture is limited by anatomical variability, whereas ultrasound guidance has shown potential to enhance puncture safety. This multicenter retrospective cohort study included 300 AF patients undergoing RFCA across 3 tertiary hospitals from September 2024 to December 2025 under uninterrupted anticoagulation. Patients were assigned to a preprocedural ultrasound localization group (n = 150) or a conventional anatomical landmark group (n = 150). Baseline characteristics were compared, and puncture efficiency, intraoperative events, puncture-related complications, operative metrics, postoperative recovery, and additional intervention requirements were evaluated. Multivariable logistic regression was used to adjust for confounders. Preprocedural ultrasound markedly improved puncture performance, achieving a higher first-attempt success rate (87.3% vs 59.3%, *P* <.001), fewer attempts (1.3 ± 0.6 vs 2.4 ± 1.1, *P* <.001), and shorter puncture time (4.6 ± 1.2 vs 6.8 ± 1.5 minutes, *P* <.001). Ultrasound-detected anatomical variations in 44% of patients, including venous deviation, duplication, stenosis, and venous–arterial overlap. Safety outcomes favored the ultrasound group, showing reduced arterial mispuncture (2.7% vs 14.0%), blood vessel spasm (3.3% vs 11.3%), and abnormal catheter path events (2.0% vs 11.3%) (all *P* <.01). Puncture-related complications were significantly decreased, including overall hematoma (10.0% vs 31.3%), persistent oozing (6.7% vs 17.3%), infection (0.7% vs 4.7%), deep vein thrombosis (2.0% vs 7.3%), pseudoaneurysm (0% vs 4.0%), and arteriovenous fistula (0.7% vs 4.7%) (all *P* <.05). Ultrasound localization also reduced operative difficulty scores (1.9 ± 0.8 vs 3.2 ± 1.1, *P* <.001) and shortened total procedural duration (118.7 ± 25.4 vs 133.3 ± 30.4 minutes, *P* <.001). Postoperative pain at 2 and 24 hours was significantly lower, and bed rest time was shorter, though length of stay was similar between groups. Additional interventions – extended compression, hematoma drainage, and anticoagulation adjustment – were markedly less frequent in the ultrasound group (overall 4.7% vs 21.3%, *P* <.001). Preprocedural femoral venous ultrasound localization significantly enhances puncture accuracy, reduces vascular complications, improves procedural efficiency, and accelerates postoperative recovery in AF patients undergoing RFCA under uninterrupted anticoagulation. These findings support incorporating ultrasound-based localization into routine preprocedural assessment to optimize the safety and quality of electrophysiological interventions.

## 1. Introduction

Atrial fibrillation (AF) is the most prevalent sustained cardiac arrhythmia worldwide, affecting more than 370 million individuals, with its incidence rising markedly with age.^[1]^ AF is strongly associated with increased risks of stroke, heart failure, cognitive decline, and all-cause mortality, underscoring the need for effective therapeutic strategies.^[2]^ Radiofrequency catheter ablation (RFCA) has become a key approach for rhythm control and is now widely used for patients with symptomatic AF or insufficient response to antiarrhythmic medications.^[3,4]^ Despite technological advances, the success and safety of RFCA continue to depend heavily on reliable vascular access, most commonly achieved via femoral venous puncture. Failed puncture or access-related complications – such as bleeding, hematoma, inadvertent arterial puncture, or deep vein thrombosis (DVT) – may reduce procedural efficiency, prolong hospitalization, and increase clinical risk.^[5]^

Current AF ablation procedures frequently employ an uninterrupted anticoagulation strategy to minimize perioperative thromboembolic events. However, vascular injury during femoral venous access under continuous anticoagulation can lead to more severe consequences. Prior studies indicate that ongoing anticoagulation significantly increases the likelihood of hematoma expansion, persistent bleeding, pseudoaneurysm formation, and arteriovenous fistula, and may complicate postoperative anticoagulation management, thereby amplifying both thrombotic and bleeding risks.^[6]^ Ensuring accurate puncture trajectory and minimizing tissue injury under uninterrupted anticoagulation has thus become a critical requirement in electrophysiology interventions.

Traditional femoral venous puncture relies on surface anatomical landmarks and operator experience, yet substantial interindividual variability – such as venous deviation, duplication, stenosis, or overlap with the femoral artery – can increase procedural difficulty.^[7,8]^ Studies have shown that landmark-guided puncture carries an inadvertent arterial puncture rate exceeding 10%, and anatomical variations often remain unrecognized without imaging guidance.^[9]^ In recent years, ultrasound guidance, whether used preprocedurally or in real time, has demonstrated clear benefits in improving central venous access success and reducing serious complications, and is already recommended in anesthesiology, emergency medicine, and critical care practice.^[10–12]^ In cardiac electrophysiology, ultrasound-based localization has gained growing attention. Evidence suggests that ultrasound allows more accurate assessment of venous position, depth, and orientation, thereby reducing arterial puncture, decreasing the number of attempts, improving patient comfort, and streamlining the procedure.^[13]^

Femoral venous access is the sole pathway for catheter entry during AF ablation, and its quality directly influences procedural safety and efficiency. Increased puncture attempts have been linked to higher rates of postoperative hematoma, greater pain, prolonged hospitalization, and potential delays in anticoagulation resumption.^[14]^ Ultrasound localization can identify vascular abnormalities in advance and guide the operator to the optimal puncture trajectory, reducing local trauma. Moreover, with rising obesity prevalence and a growing elderly AF population, the limitations of landmark-guided puncture have become increasingly evident, further highlighting the advantages of ultrasound guidance.^[15,16]^

Although existing studies suggest potential benefits of ultrasound localization, multicenter evidence specifically evaluating preprocedural femoral venous ultrasound for RFCA – particularly regarding puncture efficiency, procedural safety, and postoperative recovery – remains limited. Therefore, this study aims to compare preprocedural femoral venous ultrasound localization with conventional anatomical landmark puncture in AF patients undergoing RFCA, providing evidence to support optimization of vascular access strategies in electrophysiology practice.

## 2. Methods

### 2.1. Study design

This study was approved by the Ethics Committee of FuLing Hospital Affiliated tox Chongqing University. This multicenter retrospective cohort study included patients who underwent radiofrequency catheter ablation (RFCA) for AF under uninterrupted anticoagulation in 3 tertiary electrophysiology centers between September 2024 and December 2025. A total of 300 eligible patients were analyzed and classified into 2 groups according to whether preprocedural femoral venous ultrasound localization was performed: the ultrasound localization group (n = 150) and the anatomical landmark group (n = 150). Medical records, ultrasound images, and electrophysiology documentation were reviewed to compare puncture efficiency, procedural safety, and postoperative recovery between groups.

### 2.2. Study population

#### 2.2.1. Inclusion criteria

Patients were included if they met the following conditions: age ≥18 years; diagnosis of paroxysmal or persistent AF; RFCA performed via femoral venous access; and complete medical records, puncture documentation, and postoperative follow-up data.

#### 2.2.2. Exclusion criteria

Patients were excluded if they had congenital femoral venous anomalies, venous occlusion or severe stenosis; prior RFCA on the same side; uncontrolled bleeding risk or major coagulopathy; concurrent interventions requiring femoral venous access; or missing critical data.

#### 2.2.3. Sample size

A total of 300 patients met the criteria, with balanced enrollment across the 3 centers.

### 2.3. Group allocation and preprocedural preparation

#### 2.3.1. Ultrasound localization group

**Figure 1.**
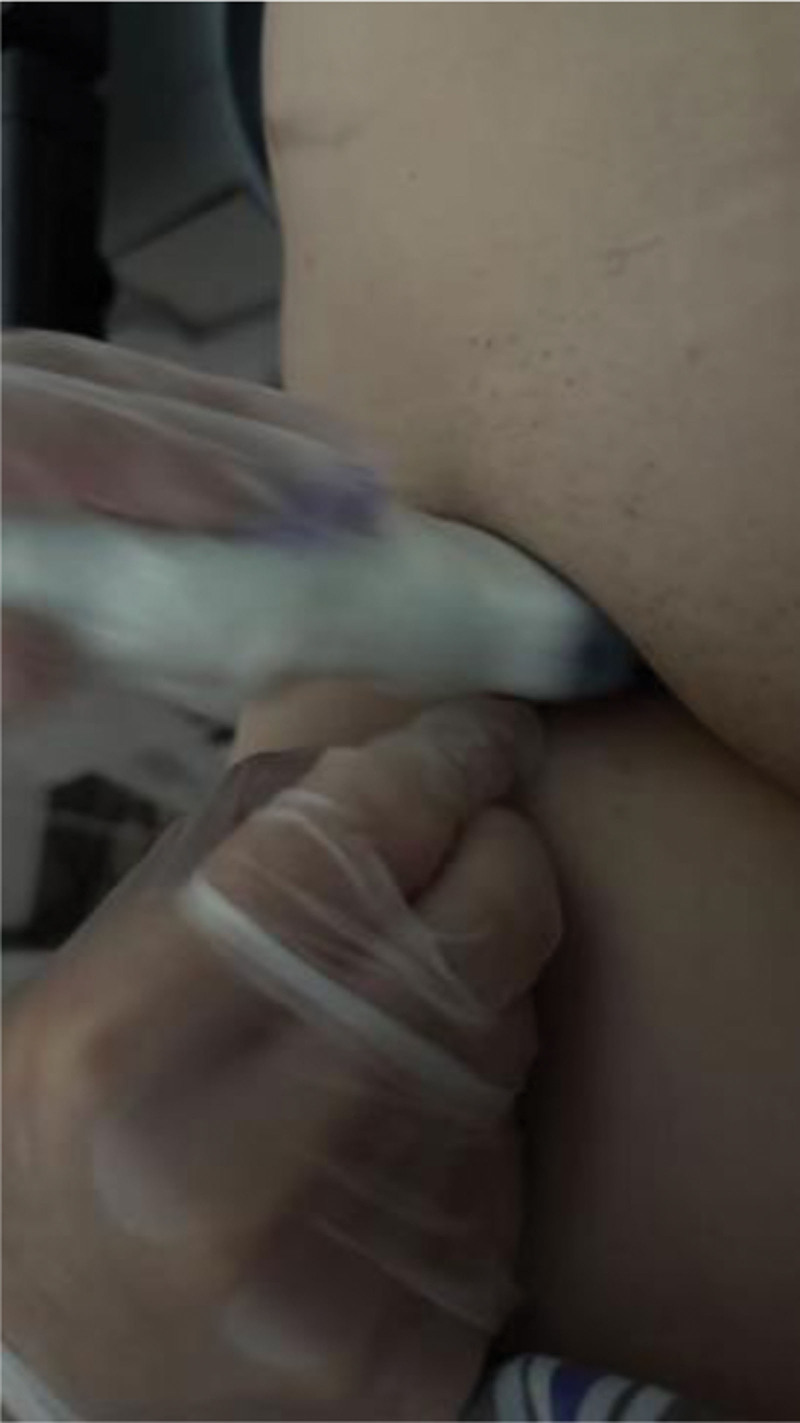
Preoperative ultrasound localization.

Before the procedure, trained cardiology operators performed transverse and longitudinal scanning of the femoral vein using a 6 to 13 MHz linear transducer. Vessel diameter, depth, trajectory, pulsatility, and flow characteristics were assessed, and the spatial relationship between the femoral vein and artery was documented. Any anatomical variations – including venous deviation, duplication, or focal stenosis – were identified, and the optimal puncture site was marked on the skin surface (Fig. 1).

#### 2.3.2. Anatomical landmark group

**Figure 2.**
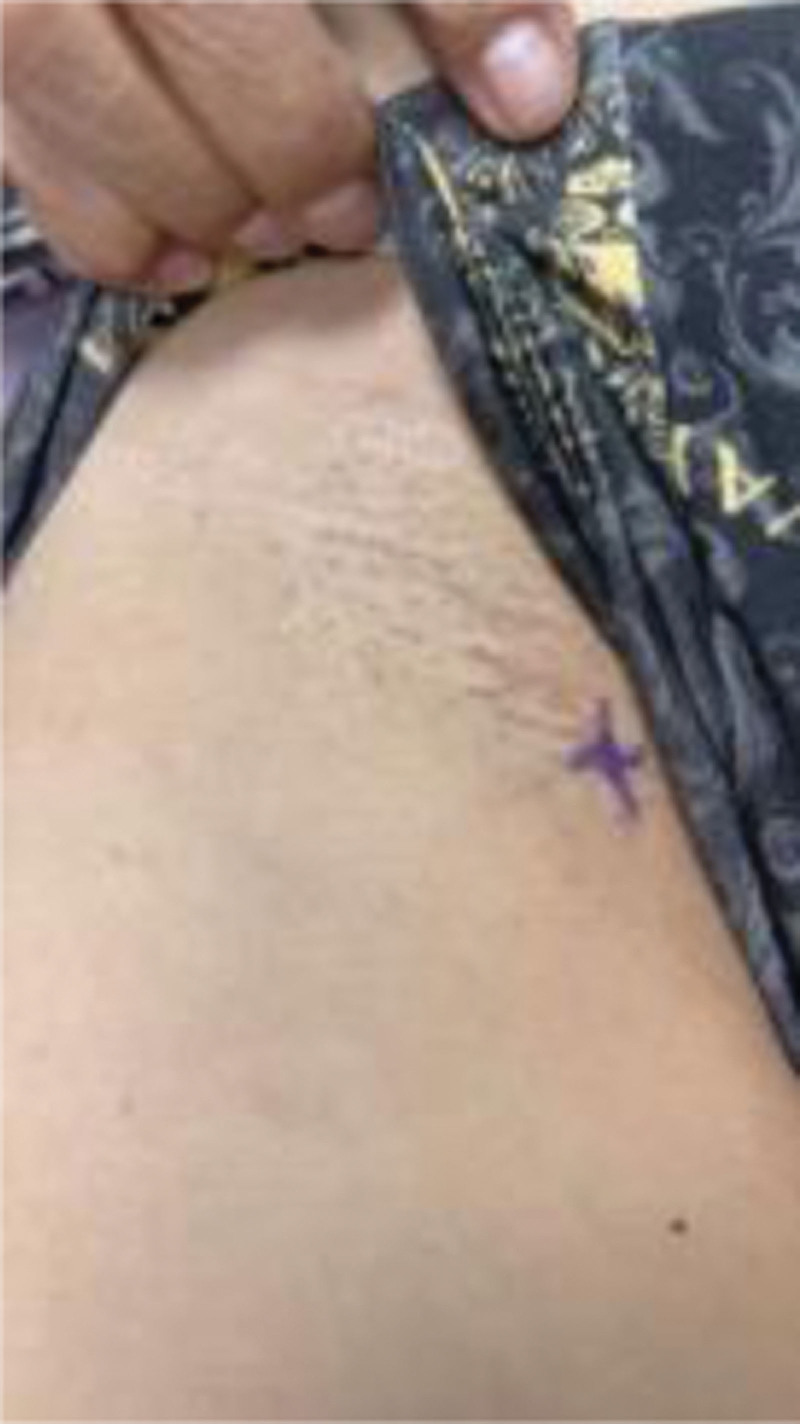
Ultrasound-guided surface marking.

**Figure 3.**
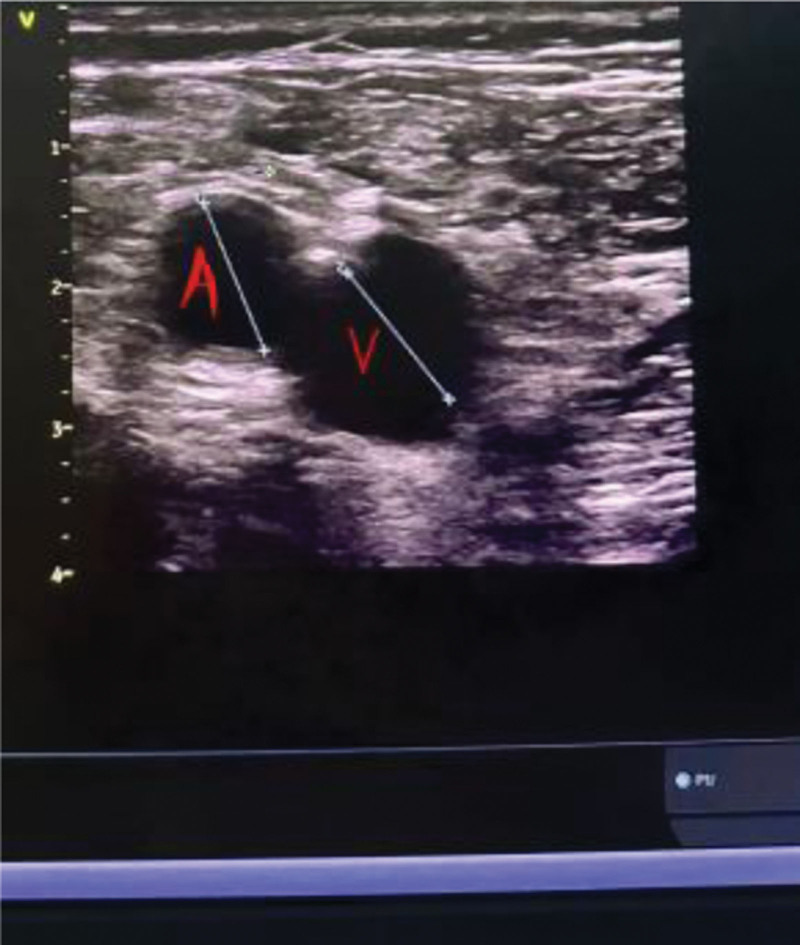
At the level of the inguinal skin fold, the femoral vein lies medial to the femoral artery.

**Figure 4.**
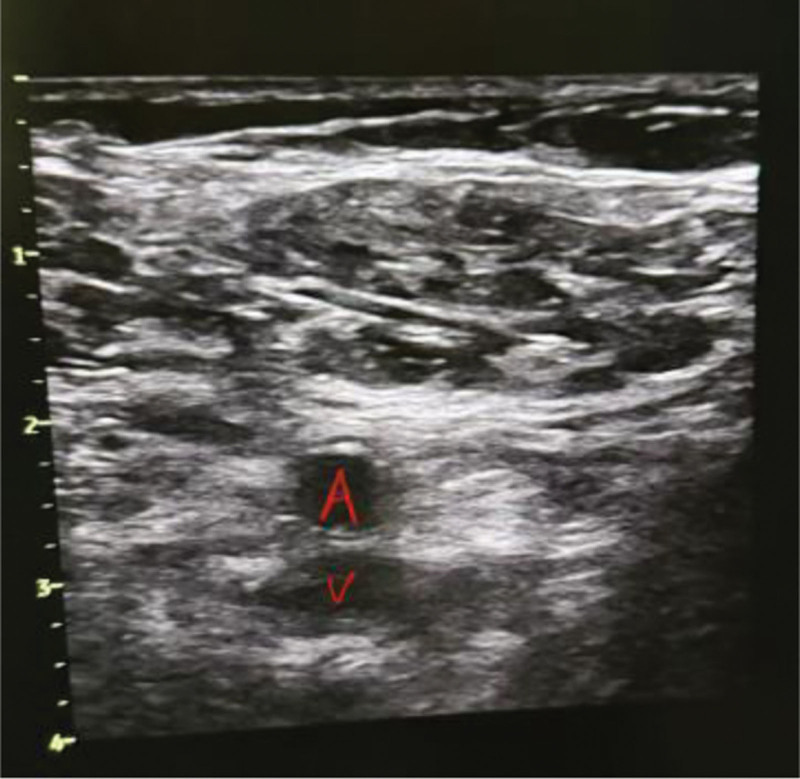
At the level of the inguinal skin fold, the femoral vein lies posterior to the femoral artery.

Patients in this group did not undergo ultrasound evaluation. Experienced electrophysiologists selected the puncture site based on palpation of the femoral arterial pulse and inguinal ligament position. Venous access was established using the standard Seldinger technique, and subsequent ablation procedures were performed identically to the ultrasound group (Figs. 2–4).

### 2.4. Outcome measures

To comprehensively assess the clinical value of preprocedural ultrasound localization, outcomes were categorized into 5 domains: puncture efficiency, puncture safety, vascular anatomy, intraoperative events, and postoperative recovery.

Key indicators included:

• first-attempt success rate, number of puncture attempts, and puncture time (reflecting accuracy and efficiency);
• ultrasound-detected anatomic features such as venous diameter, depth, flow status, and variations (e.g., deviation, duplication, stenosis);
• puncture-related safety outcomes, including arterial puncture, guidewire insertion failure or difficulty, hematoma (graded as mild/moderate/severe), persistent bleeding, DVT, pseudoaneurysm, arteriovenous fistula, and local infection – all verified through physical examination and lower-limb venous ultrasound;
• intraoperative adverse events (catheter misdirection, vascular spasm, need to switch puncture side) and operator-reported difficulty (1–5 scale);
• postoperative recovery metrics, including total procedure duration, pain scores at 2 and 24 hours (VAS), duration of bed rest, length of hospitalization, and need for additional management (e.g., hematoma intervention, prolonged compression, anticoagulation adjustments).

These multidimensional measures collectively formed a comprehensive evaluation framework for determining the impact of ultrasound localization on puncture quality, tissue injury, complication risk, and recovery.

### 2.5. Data collection

Demographic information, comorbidities, ultrasound findings, puncture records, procedural details, postoperative complications, and follow-up results were obtained from electronic medical records, ultrasound PACS, electrophysiology records, and nursing documentation. Two independent researchers performed dual data entry with consistency checks. Missing data were supplemented using available documentation where possible; unresolved missingness was flagged and accounted for in sensitivity analyses to ensure robustness.

### 2.6. Statistical analysis

All analyses were conducted using SPSS 22.0 (Chicago). Continuous variables were tested for normality using the Shapiro–Wilk test. Normally distributed data were expressed as mean ± SD and compared using the independent-samples t test; non-normal data were presented as median (IQR) and analyzed with the Mann–Whitney *U* test. Categorical variables were summarized as frequencies and percentages and compared using the chi-square test or Fisher exact test. To adjust for potential confounders (age, BMI, comorbidities, center effects), multivariable logistic regression models were constructed for key outcomes (e.g., first-attempt success, arterial puncture, hematoma), yielding adjusted odds ratios and 95% confidence intervals. The Benjamini–Hochberg method was applied to control false discovery rate across multiple safety outcomes. Sensitivity analyses were performed by excluding cases with substantial missing data or extreme values. A 2-sided *P* <.05 was considered statistically significant.

## 3. Results

### 3.1. Baseline characteristics

The 2 groups were well matched in demographic and clinical variables (Table 1). No significant differences were observed in age (63.0 ± 10.7 vs 62.1 ± 11.0 years, *P* = .492), sex distribution (male: 62.7% vs 60.0%, *P* = .603), BMI (24.2 ± 3.3 vs 23.9 ± 3.6 kg/m^2^, *P* = .458), or other baseline parameters including hypertension, diabetes, AF type, left atrial diameter, and LVEF (all *P* >.05). These findings confirm balanced preprocedural profiles, ensuring comparability for subsequent analyses.

**Table 1:** Baseline characteristics of the 2 groups.

Variable	Ultrasound localization (n = 150)	Anatomical landmark (n = 150)	Statistic (t/χ^2^)	*P*-value
Age (years)	63.0 ± 10.7	62.1 ± 11.0	0.69	.492
Male sex (n [%])	94 (62.7%)	90 (60.0%)	0.27	.603
BMI (kg/m^2^)	24.2 ± 3.3	23.9 ± 3.6	0.74	.458
Hypertension (n [%])	77 (51.3%)	82 (54.7%)	0.36	.55
Diabetes (n [%])	44 (29.3%)	41 (27.3%)	0.15	.695
AF type (persistent) (n [%])	72 (48.0%)	68 (45.3%)	0.21	.647
Left atrial diameter (mm)	41.9 ± 5.7	42.4 ± 5.3	0.77	.441
LVEF (%)	58.1 ± 6.3	58.4 ± 6.0	0.43	.667

AF = atrial fibrillation, BMI = body mass index, LVEF = left ventricular ejection fraction.

### 3.2. Ultrasound assessment of vascular anatomy

Preprocedural scanning in the ultrasound localization group revealed multiple anatomical variations (Table 2). Venous duplication was present in 12.0% of patients, venous deviation in 14.7%, slow venous flow in 19.3%, and substantial overlap with the femoral artery in 10.0%. Additionally, 7.3% showed varying degrees of stenosis. These observations demonstrate that ultrasound markedly enhances the detection of anatomical abnormalities that are often missed with landmark-guided puncture, and which may contribute to puncture difficulty or complications.

**Table 2:** Ultrasound anatomic findings.

Parameter	Ultrasound group	Abnormality rate (%)
Femoral vein duplication	18	12.00
Femoral vein deviation (>5 mm)	22	14.70
Femoral vein stenosis (≥30%)	11	7.30
Slow venous flow	29	19.30
Vein–artery overlap	15	10.00

### 3.3. Puncture efficiency

Puncture performance differed significantly between groups (Table 3). The ultrasound group achieved a higher first-attempt success rate (87.3% vs 59.3%, *P* <.001), required fewer attempts (1.3 ± 0.6 vs 2.4 ± 1.1, *P* <.001), and demonstrated shorter total puncture time (4.6 ± 1.2 vs 6.8 ± 1.5 minutes, *P* <.001). Guidewire insertion difficulty was also markedly reduced (4.0% vs 18.7%, *P* <.001).

**Table 3:** Puncture efficiency indicators.

Parameter	Ultrasound Group	Anatomical Landmark Group	Statistic	*P*-value
First-attempt success (n [%])	131 (87.3%)	89 (59.3%)	30.86	<.001
Number of puncture attempts	1.3 ± 0.6	2.4 ± 1.1	9.88	<.001
Puncture time (min)	4.6 ± 1.2	6.8 ± 1.5	11.12	<.001
Guidewire insertion difficulty (n [%])	6 (4.0%)	28 (18.7%)	16.13	<.001

### 3.4. Intraoperative safety events

The ultrasound localization group exhibited substantially fewer intraoperative adverse events (Table 4). Rates of inadvertent arterial puncture (2.7% vs 14.0%, *P* <.001), vascular spasm (3.3% vs 11.3%, *P* = .007), and abnormal catheter trajectory (2.0% vs 11.3%, *P* = .001) were all significantly lower. The need to switch puncture sides was also reduced (2.0% vs 8.7%, *P* = .008). These results indicate that ultrasound improves procedural safety by enabling more accurate vessel targeting.

**Table 4:** Intraoperative safety events.

Parameter	Ultrasound group	Anatomical landmark group	Statistic	*P*-value
Inadvertent arterial puncture (n [%])	4 (2.7%)	21 (14.0%)	14.31	<.001
Vascular spasm (n [%])	5 (3.3%)	17 (11.3%)	7.19	.007
Switch of puncture side (n [%])	3 (2.0%)	13 (8.7%)	7.05	.008
Abnormal catheter trajectory (n [%])	3 (2.0%)	17 (11.3%)	10.36	.001

### 3.5. Puncture-related complications

Complications related to venous access showed clear between-group differences (Table 5). Overall hematoma incidence was notably lower in the ultrasound group (10.0% vs 31.3%, *P* <.001), with fewer moderate-to-severe events. Persistent oozing (6.7% vs 17.3%, *P* = .007) and puncture-site infection (0.7% vs 4.7%, *P* = .030) were likewise less frequent. DVT occurred more often in the landmark group (7.3% vs 2.0%, *P* = .027).

**Table 5:** Puncture-related complications.

Parameter	Ultrasound group (n = 150)	Anatomical landmark group (n = 150)	Statistic	*P*-value
Overall hematoma (n [%])	15 (10.0%)	47 (31.3%)	19.46	<.001
Mild	12	28	–	–
Moderate	3	16	–	–
Severe	0	3	–	–
Persistent oozing (n [%])	10 (6.7%)	26 (17.3%)	7.33	.007
Puncture-site infection (n [%])	1 (0.7%)	7 (4.7%)	4.69	.03
DVT (n [%])	3 (2.0%)	11 (7.3%)	4.92	.027
Pseudoaneurysm (n [%])	0 (0.0%)	6 (4.0%)	6.11	.013
AV fistula (n [%])	1 (0.7%)	7 (4.7%)	4.69	.03

AV = atrioventricular, DVT = deep vein thrombosis.

**Figure 5.**
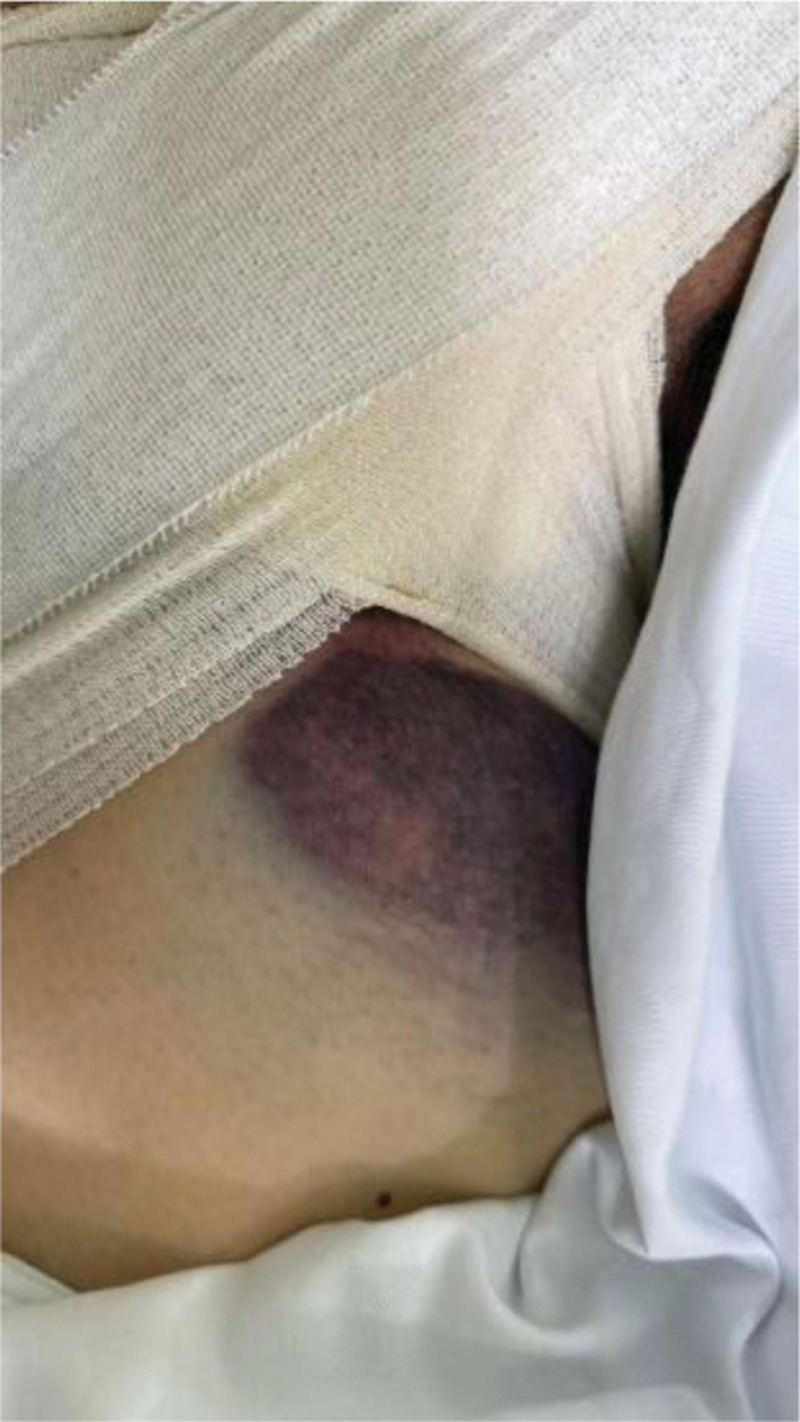
Subcutaneous hematoma at the right femoral vein puncture site.

**Figure 6.**
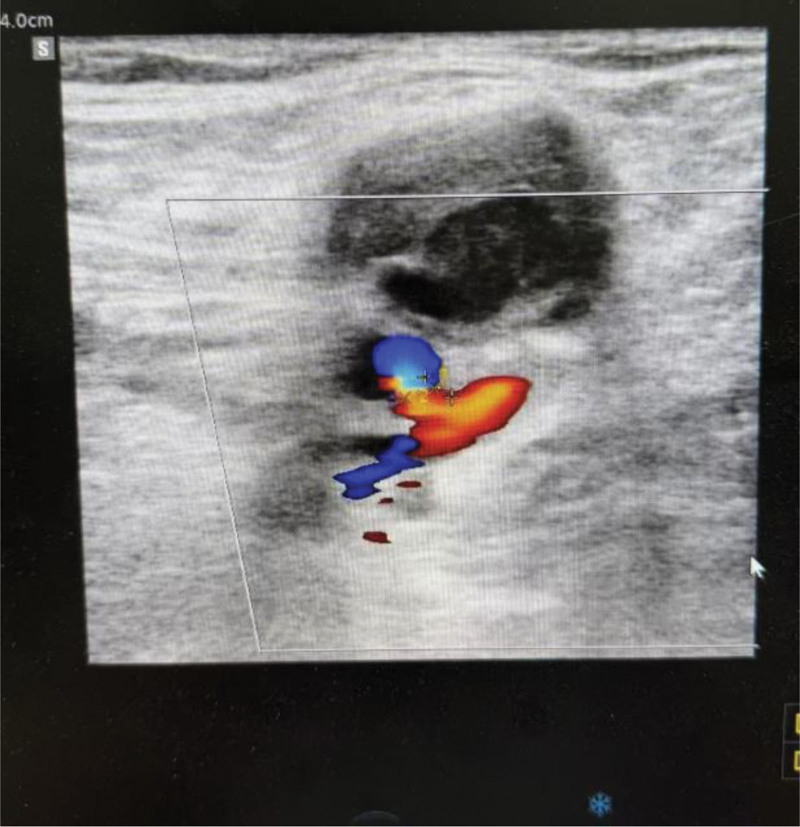
Arteriovenous fistula with pseudoaneurysm formation at the right femoral vein puncture site.

Severe vascular complications also favored the ultrasound group: pseudoaneurysm occurred only in the landmark group (4.0% vs 0%, *P* = .013), and arteriovenous fistula was markedly more common (4.7% vs 0.7%, *P* = .030). These findings reinforce that ultrasound reduces vessel-wall injury caused by mispunctures or repeated attempts. (Figs. 5 and 6)

### 3.6. Procedural characteristics

Operator-reported difficulty scores were significantly lower in the ultrasound group (1.9 ± 0.8 vs 3.2 ± 1.1, *P* <.001), reflecting easier and more predictable access (Table 6). Total procedure duration was also shorter (118.7 ± 25.4 vs 133.3 ± 30.4 minutes, *P* <.001), suggesting improved efficiency and potentially reduced exposure to anesthesia and procedural risk.

**Table 6:** Operative characteristics.

Parameter	Ultrasound group	Anatomical landmark group	Statistic	*P*-value
Operator difficulty score (1–5)	1.9 ± 0.8	3.2 ± 1.1	10.23	<.001
Total procedure duration (min)	118.7 ± 25.4	133.3 ± 30.4	4.19	<.001

### 3.7. Postoperative recovery

Ultrasound localization resulted in more favorable recovery outcomes (Table 7). Pain scores at 2 and 24 hours post-procedure were significantly lower (both *P* <.001), and bed rest time was reduced (5.1 ± 1.1 vs 6.5 ± 1.4 hours, *P* <.001). Although hospitalization duration was similar between groups (*P* = .201), the overall pattern indicates accelerated early recovery with less procedural trauma.

**Table 7:** Postoperative recovery indicators.

Parameter	Ultrasound group	Anatomical landmark group	Statistic	*P*-value
VAS (2 h)	2.3 ± 1.0	3.7 ± 1.3	9.11	<.001
VAS (24 h)	1.4 ± 0.8	2.3 ± 1.0	8.19	<.001
Bed rest duration (h)	5.1 ± 1.1	6.5 ± 1.4	8.12	<.001
Length of stay (d)	3.7 ± 1.1	3.9 ± 1.3	1.28	.201

VAS = visual analogue scale.

### 3.8. Need for additional interventions

The landmark group required substantially more postprocedural management (Table 8). Prolonged compression (15.3% vs 3.3%, *P* <.001), ultrasound-guided hematoma treatment (6.0% vs 0.7%, *P* = .014), and anticoagulation adjustments (8.7% vs 2.7%, *P* = .028) were all more frequent. Overall reintervention rates were significantly higher in the landmark group (21.3% vs 4.7%, *P* <.001). These results indicate that preprocedural ultrasound not only improves immediate safety but also reduces downstream clinical workload and resource utilization.

**Table 8:** Need for additional interventions.

Parameter	Ultrasound group	Anatomical landmark group	Statistic	*P*-value
Prolonged compression (n [%])	5 (3.3%)	23 (15.3%)	12.56	<.001
Ultrasound-guided hematoma management (n [%])	1 (0.7%)	9 (6.0%)	6.06	.014
Anticoagulation adjustment (n [%])	4 (2.7%)	13 (8.7%)	4.8	.028
Overall reintervention (n [%])	7 (4.7%)	32 (21.3%)	19.21	<.001

## 4. Discussion

This multicenter retrospective study comprehensively compared preprocedural femoral venous ultrasound localization with conventional anatomical landmark puncture in patients undergoing RFCA for AF. The findings demonstrate that ultrasound localization significantly improves first-attempt success, reduces the number of puncture attempts and total puncture time, and markedly lowers the incidence of arterial mispuncture, hematoma, and other access-related complications. Improvements were also observed in postoperative pain, bed rest duration, and overall recovery. Collectively, these data highlight the substantial value of incorporating ultrasound-based assessment into AF ablation workflows to enhance procedural safety and efficiency.

The markedly higher first-attempt success rate in the ultrasound group aligns with findings from previous electrophysiology studies. Sobolev et al reported that ultrasound-guided femoral venous access substantially decreases initial puncture failure and repeat attempts.^[17]^ Subsequent systematic reviews reinforced that ultrasound guidance shortens puncture time and reduces the number of attempts compared with landmark-based techniques.^[18,19]^ This benefit is largely attributable to real-time visualization of vessel position, depth, and trajectory, which mitigates misalignment caused by obesity, advanced age, or poorly defined surface landmarks. Wiles et al even recommended ultrasound as the “new standard” for establishing vascular access in EP laboratories.^[20]^ Our multicenter real-world results further support this position.

Ultrasound screening in the present study identified venous deviation, duplication, focal stenosis, and vein–artery overlap in a considerable proportion of patients – findings consistent with the anatomical variation rates described by Guan et al.^[21]^ Prior work has established that such variations are important contributors to puncture difficulty and inadvertent arterial entry during landmark-guided access.^[22]^ Yamagata et al also showed that unrecognized vascular anomalies are associated with increased vascular complications during AF ablation.^[23]^ By revealing these variants in advance, ultrasound enabled operators to avoid suboptimal access sites, explaining the higher puncture success and lower guidewire placement difficulty observed in the ultrasound group.

Regarding safety outcomes, ultrasound localization was associated with a significant reduction in arterial mispuncture and access-site hematoma. A landmark BMJ meta-analysis by Hind et al demonstrated that ultrasound-guided venous puncture reduces arterial puncture risk by approximately 70%,^[24]^ with similar conclusions reported by Cochrane reviews.^[19]^ In the context of AF ablation, access-site hematoma has particular clinical relevance: Piccini et al noted that hematoma prolongs hospitalization and can complicate anticoagulation management, escalating both thrombotic and hemorrhagic risks.^[25]^ Sharma et al similarly observed fewer groin complications when ultrasound was used during AF ablation.^[26]^ The safety benefits observed in our study are highly consistent with these prior findings.

Ultrasound localization also had favorable implications for the procedural workflow. The total ablation duration was shorter in the ultrasound group, echoing observations from Kirkfeldt et al, who showed that vascular complications are an important driver of prolonged EP procedures and higher healthcare costs.^[27]^ Reduced operator difficulty scores further suggest that ultrasound simplifies access and may enhance training efficiency – a conclusion supported by Powell et al, who demonstrated improved procedural experience among less-experienced physicians using ultrasound-guided venous puncture.^[28]^

Postoperative recovery metrics likewise favored the ultrasound group. Lower pain scores and shorter bed rest duration are consistent with Brattain et al, who found a positive correlation between puncture attempts and local pain severity.^[29]^ Minimizing tissue damage not only accelerates mobilization but may also improve patient satisfaction. Although international guidelines do not mandate ultrasound use for AF ablation, they increasingly emphasize the importance of preventing vascular complications.^[30,31]^

Particularly notable in this study is the reduced incidence of pseudoaneurysm and arteriovenous fistula – less common but clinically serious complications – in the ultrasound group. Pseudoaneurysm typically results from arterial wall injury and may expand or rupture under uninterrupted anticoagulation, sometimes requiring surgical intervention.^[32,33]^ Arteriovenous fistula often arises from inadvertent arterial puncture or guidewire misplacement and may lead to bruit, venous congestion, or increased cardiac preload.^[34,35]^ Given that AF ablation frequently employs uninterrupted anticoagulation, such complications pose heightened risk, including hematoma expansion and repeated bleeding.^[6]^ By accurately identifying the relative positions and overlap of the femoral artery and vein, ultrasound minimizes both mispuncture and repeated attempts, thereby reducing serious downstream complications. Real-world EP laboratory analyses and multiple systematic reviews have similarly shown that ultrasound guidance reduces arterial puncture by 50% to 80% and nearly eliminates subsequent pseudoaneurysm formation.^[20,36]^ Our results reinforce these observations and underscore the importance of ultrasound in preventing high-risk vascular events, especially under continuous anticoagulation.

Using data from multiple centers, this study provides robust evidence supporting preprocedural femoral venous ultrasound as a strategy to improve access success, enhance safety, and optimize recovery in AF ablation. Nonetheless, several limitations merit consideration. First, the retrospective design introduces the possibility of selection bias. Second, the study did not distinguish the relative benefits of static preprocedural ultrasound versus real-time ultrasound guidance. Third, the sample size may have been insufficient to detect differences in rare complications. Prospective randomized trials are needed to further validate these findings.

In summary, preprocedural femoral venous ultrasound localization significantly improves puncture success and safety, reduces vascular complications, and facilitates postoperative recovery in patients undergoing RFCA for AF. When feasible, this technique should be integrated into routine practice as an essential component of contemporary AF ablation workflows.

## Author contributions

**Conceptualization:** Xiaobo Liao, Qiao Xiao, Xueyuan Guo, Yunong Li, Xin Chen, Longjiang Hu, Xin Su, Lan Ren, Fangbing Wang, Peng Xiao.

**Data curation:** Xiaobo Liao, Qiao Xiao, Xueyuan Guo, Yunong Li, Xin Chen, Longjiang Hu, Xin Su, Lan Ren, Fangbing Wang, Peng Xiao.

**Formal analysis:** Xiaobo Liao, Qiao Xiao, Xueyuan Guo, Yunong Li, Xin Chen, Longjiang Hu, Xin Su, Lan Ren, Fangbing Wang, Peng Xiao.

**Funding acquisition:** Xiaobo Liao, Qiao Xiao, Yunong Li, Xin Chen, Longjiang Hu, Fangbing Wang, Peng Xiao.

**Investigation:** Xiaobo Liao, Peng Xiao.

**Writing** – **original draft:** Peng Xiao.

**Writing** – **review & editing:** Peng Xiao.
